# Round the Hospitals

**Published:** 1917-07-14

**Authors:** 


### ROUND THE HOSPITALS.

A meeting was held, by the courtesy of the Guardians and the matron, at the Whitechapel Infirmary, Vallance Road, on Monday evening, when Miss Rundle spoke to a very representative and interested gathering upon the objects and progress of the College of Nursing. Miss Mowat, the matron, took the chair. There were present, besides members of the staff, Miss Stewart, matron of the City of London Infirmary; Miss Ingles, matron of Shoreditch Infirmary; Miss Dalton, matron of the Victoria Park Chest Hospital, with sisters and nurses representing these training-schools; also nurses representing King's College Hospital and district nursing. The nurses were very responsive, and asked many questions. Miss Mowat asked the nurses not to think of the College as just another Association to belong to, but to think of it in its broad, comprehensive nature, which would influence the profession all over the British Empire. Refreshments were kindly provided for the visitors. Miss Rundle hopes to attend meetings in Nottingham and Hull shortly.

The Central Committee is pursuing its policy of suicide so far as any claim it may have had to possess public interest or to be a public organisation. Having begun in a roundabout way to try to eject the two most important societies represented on the Central Committee—*i.e.*, the Royal British Nurses' Association and the Association for the Promotion of the Registration of Nurses in Scotland—it recently excluded the press from its meetings. We hope that matrons, sisters, and nurses will note this fact, which may indicate the destruction of any influence it ever had by excluding reporters and supplying *ex parte* reports of its proceedings at the will of a small clique who control its policy. We expect that the independent societies represented on this Committee will protest against the exclusion of the press in future, for this step would tend to destroy confidence in its proceedings, and reduce them to impotence so far as Parliament, the profession, and the public are concerned.

Recently a happy idea found expression with generous prodigality through the kindly forethought of some women governors of St. Mary's Hospital, the Misses Leventhorpe. Taking advantage of the strawberry season, these ladies generously provided a strawberry tea for the nursing staff at St. Mary's Hospital, Paddington, and no efforts were spared to make the occasion memorable for its cordiality and pleasurable enjoyment. The nursing staff from the youngest probationer to the eldest sister were present. The idea is one which might find many imitators, especially when, as at St. Mary's, the banqueting chamber was made beautiful with plants and flowers, and the matron, the home sister, and the assistant matron, who are respectively, Miss Jackson, Miss Morrison, and Miss Mehew, with the Misses Leventhorpe, made charming hostesses and most successful entertainers. Altogether the gathering at this strawberry feast will stand out as one of the happiest of the year. The success of this new departure in hospital entertainments warrants its perpetuation as an annual feast for the nursing staff of every well-administered hospital throughout the country. In wartime it would be a graceful act for the fortunate possessors of well-cultivated gardens to give spontaneously a number of strawberry or fruit teas in the next fortnight or three weeks to the nursing staff of some London or provincial hospital, or to both. Flowers for the decorations would be welcome companions to the fruit.

